# Association of Maximum Temperature With Sedentary Time in Older British Men

**DOI:** 10.1123/jpah.2016-0468

**Published:** 2016-12-29

**Authors:** Claudio Sartini, Richard W Morris, Peter H Whincup, S Goya Wannamethee, Sarah Ash, Lucy Lennon, Barbara J Jefferis

**Affiliations:** Dept of Primary Care & Population Health; University College London, UK; UCL Physical Activity Research Group (PARG) University College London, UK; Dept of Primary Care & Population Health; University College London, UK; School of Social and Community Medicine, University of Bristol, Bristol, UK; Population Health Research Institute, St George’s University of London, London, UK; Dept of Primary Care & Population Health University College London, UK; Dept of Primary Care & Population Health; University College London, UK; UCL Physical Activity Research Group (PARG) University College London, UK

**Keywords:** sedentary behavior, older adults, weather, epidemiology, accelerometry

## Abstract

**Background:**

Sedentary behavior is very common in older adults and a risk factor for mortality. Understanding determinants of sedentary behavior may help in defining strategies aimed to reduce the time spent sedentary. The degree of difference in sedentary time attributable to varying temperatures has not been yet estimated in older men.

**Methods:**

Men aged 71 to 91 years participating in an established UK population-based cohort study were invited to wear an Actigraph GT3X accelerometer for 1 week in 2010–12. Outcome was sedentary time (<1.5 Metabolic Equivalent of Task) in minutes per day. Associations between daily outdoor maximum temperature and accelerometer-measured sedentary time were estimated using multilevel models.

**Results:**

43% (1361/3137) of invited men participated in the study and provided adequate data. Men spent on average 615 minutes in sedentary time per day (72% of the total accelerometer-wear time). After adjusting for covariates, men spent 26 minutes more per day (*P* < .001) in sedentary time when temperatures were in the lowest (–3.5; 9.2°C) versus highest quintile (19.1; 29.5°C).

**Conclusions:**

Sedentary time in older adults is highest at lowest temperatures, typically recorded in winter. Findings are relevant for guidelines: interventions may consider targeting older men in winter providing recommendations for minimizing sedentariness on daily basis.

A standard definition of sedentary behavior has not yet been established, although contemporary researchers agree that sedentary behavior is not simply a lack of physical activity.^1^ Sedentary behavior can be defined as the time spent in activities engendering less than 1.5 Metabolic Equivalent of Task (METs).^2^ In recent years, there have been an increasing number of studies which have reported associations between prolonged sedentary behavior and health outcomes, such as mortality and cardiovascular disease, which have been independent of physical activity levels.^3^ Therefore, understanding determinants of sedentary behavior may help in defining strategies aimed to reduce the time spent sedentary. This is particularly important in older adults, who are known as the most sedentary of all age-groups.^4^

A few previous studies in older adults have demonstrated that low outdoor temperatures were associated with less time spent in physical activity,^5^,^6^ although an association with sedentary time was not investigated. We would intuitively expect sedentary time to be higher at lower temperatures, as occur during the winter season. However, the degree of difference in sedentary time attributable to varying outdoor temperatures has not been estimated in previous studies of older adults. Outdoor temperature has been overlooked in sedentary behavior guidelines,^7^ and as determinant of sedentary time.^8^,^9^ To our knowledge an association of temperature with sedentary time in older adults has not been previously documented.

Considering the gaps in knowledge of previous research, we have therefore investigated how sedentary time (<1.5 METs) varies according to outdoor maximum temperature in a large UK population based cohort study of community-dwelling older men.

## Methods

### Participants

The British Regional Heart Study (BRHS) is a prospective cohort of men recruited from a single local primary care center in 24 British towns in 1978–80.^10^ In 2010–2012, the surviving participants resident in the United Kingdom (UK), then aged 71 to 91, were invited to attend a further physical examination and to participate in a study of objectively measured physical activity, on which the analyses presented here are based. Men who met the inclusion criteria (not living in a residential home and not being on wheelchair) were included. Participants completed a log diary (detailing when the accelerometer was worn) and a comprehensive health status questionnaire. The participants’ individual characteristics and questionnaire data were already described elsewhere.^11^ The National Research Ethics Service Committee for London provided ethical approval. Participants provided informed written consent to the investigation, which was performed in accordance with the Declaration of Helsinki.

### Measurements and Data Analysis

Repeated measures of physical activity levels per each participant were recorded over the course of 1 week by using accelerometers. Methods for accelerometer-data extraction and processing were previously described in detail,^11^ and added here as supplementary material (see Online Supplementary Material, Appendix S1). In brief, the number of minutes per day in spent in sedentary behavior, light physical activity (LIPA) and moderate-to-vigorous physical activity (MVPA) was derived and categorized using count-based intensity threshold values of counts per minute (CPM) developed for older adults, as in previous studies; the cut-points used were <100, 100 to 1040, >1040 CPM for sedentary time (<1.5 METs), time spent in LIPA (<1.5 to 2.9 METs) and MVPA (≥3 METs) respectively.^4^,^12^,^13^ Number of steps per day was also recorded as a measure of overall physical activity. Then, maximum temperatures were linked to the accelerometer data for each day the men wore the device. Daily temperatures (maximum and minimum), hours of sunshine, and relative humidity were provided by the UK Meteorological (MET) Office (see Online Supplementary Material, Appendix S1). Maximum temperature was used as the main exposure variable and divided into quintiles. Quintiles were chosen as temperatures in the lowest quintile (1Q, –3.5°C; 9.2°C) were representative of the typical UK winter, while temperatures in highest quintile (5Q: 19.1°C; 29.5°C) were representative of the typical UK summer.^14^ The main outcome investigated was sedentary time measured in minutes per day. In preliminary analysis, the correlations between sedentary time and other PA variables (steps, LIPA, and MVPA) were calculated. Linear multilevel models (level 1 was the date of wear and level 2 was the individual) with random intercept only were used to estimate associations between quintiles of maximum temperature and sedentary time. Quintiles of maximum temperature were derived counting every day each participant wore an accelerometer. The highest quintile of maximum temperature (5th quintile, 5Q) was chosen as reference quintile, and the results were reported as mean difference in sedentary time between the reference vs lower quintiles. As in one previous study,^11^ the model was adjusted for measurement variables [accelerometer-wear time, wear day order (first day of wear, second, etc), day of the week, age, social class, Body Mass Index (BMI), chronic conditions, mobility limitations, geriatric depression scale, vision problems, smoking status, and day length (a proxy for season)]. The adjustment for day length was made to check whether there was confounding between temperature and a different seasonal term (collinearity was not observed as the Variance Inflation Factor (VIF) score was less than 1.5). As sensitivity analysis, a linear model was performed using maximum temperature as a continuous variable instead of the quintiles.

### Subsidiary Analyses

For completeness of information, we investigated associations of maximum temperature with different outcomes related to sedentary behavior: total number of sedentary breaks per day, daily number of sedentary bouts of <30 minutes, and daily number of sedentary bouts of ≥30 minutes.

Associations of minimum temperature, hours of sunshine, and relative humidity (continuous variables) with sedentary time were also estimated.

A further investigation was performed to corroborate findings from previous studies which made use of different physical activity outcomes, rather than sedentary time. Therefore, associations of temperatures (maximum and minimum), hours of sunshine, and relative humidity with daily (i) number of steps, (ii) minutes spent in LIPA, and (iii) minutes spent in MVPA were estimated.

We also performed stratified analysis by excluding men who were depressed or/and with mobility limitations. All analyses were carried out using STATA/SE 13^15^ and MLwiN Version 2.30.^16^

## Results

1455 (46%) surviving men participated and met the inclusion criteria. 1361 men (43.4%) had data on all covariates (complete case analysis) and they had same mean age (78.5 years, SD = 4.6) and BMI (26.7, SD = 3.3) in comparison with 1455 men who met the inclusion criteria. The 1361 men with complete data were used in the final analysis: men had a mean of 6.5 (SD = 1.2) valid days of accelerometer wear; they wore the accelerometer for 855 minutes per day (SD = 93) and took on average 4872 steps per day (SD = 2767). The average sedentary time per day was 615 minutes (SD = 83), corresponding to 72% of the total accelerometer wear time; time spent in LIPA and MVPA was 198 minutes (SD = 65) and 39 minutes (SD = 32) respectively. The correlations between daily sedentary time with steps, LIPA, and MVPA were –0.46, –0.54, and –0.47 respectively (all *P*-values < 0.001).

### Descriptive Statistics

**Figure 1:**
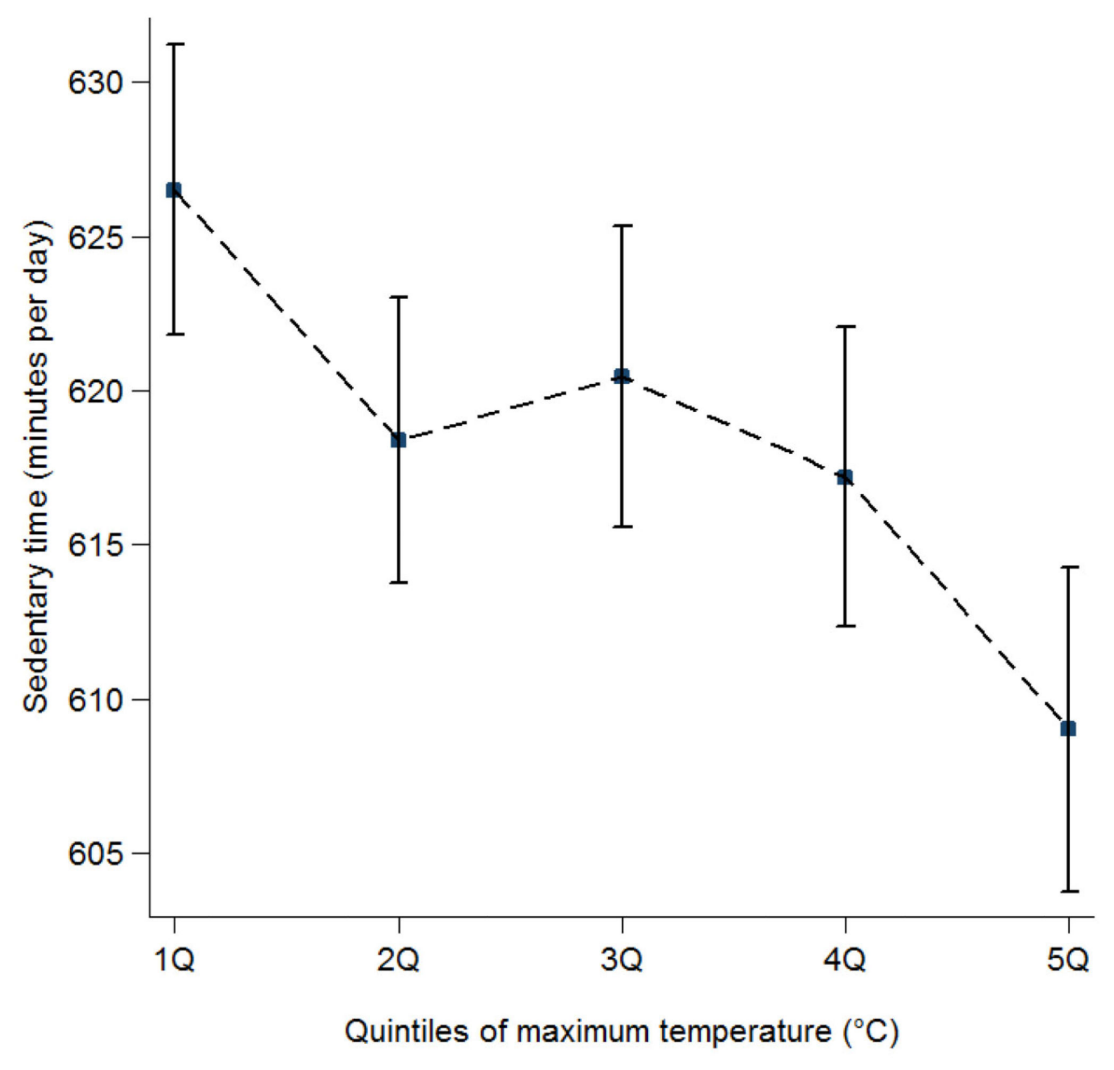
Raw data (n = 1361). Plots depicting relationship between sedentary time (mean, 95% CI), and quintiles (Q) of maximum temperature. *Note.* Quintiles of maximum temperature were derived counting every day each participant wore an accelerometer (median, minimum and maximum): 1Q: 6.3 (–3.5, 9.2); 2Q: 11.0 (9.3, 13.0); 3Q: 15.3 (13.1, 16.5); 4Q: 17.9 (16.6, 19.0); 5Q: 20.8 (19.1, 29.5). *P*-value for the difference between the quintiles was *P* < .001.

The median for maximum temperature in the lowest quintile was 6.3°C (between -3.5°C and 9.2°C) and in highest quintiles was 20.8°C (between 19.1°C and 29.5°C). In descriptive plots, unadjusted sedentary time was highest when temperatures were in the lowest quintiles, and then decreased at higher temperatures (Figure 1).

### Associations Between Maximum Temperatures and Sedentary Time

The adjusted associations from multilevel models between quintiles of maximum temperature and sedentary time are shown in Table 1. In summary, lower temperatures were associated with more time spent in sedentary behavior (*P* < .001). In particular, men spent 26 minutes more per day (95% CI 19–33) in sedentary time when temperatures were in the lowest compared with the highest quintile (Table 1). When analyzing maximum temperature as continuous variable, a negative linear association with sedentary time was observed: a decrease in 1 SD (5.8°C) in maximum temperature was associated with an increase of 11 minutes per day (95% CI 8–13) in sedentary time (*P* < .001). The adjustment for day length did not alter the magnitude of these associations; day length was not significantly associated with sedentary time (*P* = .212).

### Subsidiary Analyses

A decrease of 1 SD (5.8°C) in maximum temperature was associated with a decrease of 2 (95% CI 1–3) breaks in sedentary time per day, and an increase of 0.2 (95% CI 0.1–0.3) daily number of longer sedentary bouts (≥30 minutes). No association was found between maximum temperature and daily number of shorter sedentary bouts (<30 minutes).

Variations in hours of sunshine and relative humidity were associated with variations in sedentary time. On the other hand, association of minimum temperature with sedentary time was not significant (Online Supplementary Material, Appendix S1, Table 1).

Maximum temperature was also strongly associated with other physical activity outcomes: a decrease of 1 SD in maximum temperature was associated with –7 minutes in LIPA per day (95% CI –9 to –5), –4 minutes in MVPA per day (95% CI –5 to –2), and –323 steps per day (95% CI –428 to –218). Similarly, variations of hours of sunshine and relative humidity were also associated with variations of time spent in LIPA, MVPA, and steps per day, although the magnitude of associations was smaller. Association of minimum temperature with physical activity was not significant (Online Supplementary Material, Appendix S1, Table 1).

In stratified analysis, the magnitude of associations between temperature and sedentary time were not materially affected by excluding men who were depressed or/and with mobility limitations (Online Supplementary Material, Appendix S1, Table 2).

## Discussion

In this large study of older British men, outdoor maximum temperature was associated with accelerometer-measured sedentary time: a decrease in maximum temperatures was associated with an increase in sedentary time after controlling for potential confounding variables (measurement variables, individual characteristics, and day length).

### Overall Findings

The analysis of maximum temperature subdivided in quintiles offered a simple and intuitive interpretation of the results: during a typical winter day (temperature in the lowest quintile) older men spent 26 minutes more per day in sedentary time in comparison with a typical summer day (temperatures in the highest quintiles). Perception of cold may particularly inhibit older individuals from spending time outdoors. Apart from the discomfort and need to wear suitable clothing, there may be a fear of falling due to ice. Consequently, older adults may prefer replacing some incidental light physical activity outdoors (eg, a gentle walk for pleasure) with sedentary behaviors indoors, such as television watching.^17^

We focused our investigation on maximum temperature as primary determinant as it is more accurate than other meteorological factors due to a lower spatial variability.^18^ However, in subsidiary analysis we also demonstrated that less hours of sunshine and higher relative humidity, typical elements of the winter season in UK, were also associated with an increase in sedentary time. To our knowledge these findings are novel and not previously reported. Literature in this field is sparse; 1 small study of 46 adults demonstrated that accelerometer-measured sedentary time is higher in winter than summer, although the participants were about 40 years younger than our population.^19^ The majority of the studies investigated children or adolescents, which are known to have a different life-style in comparison with older adults.^20^

### Strengths and Limitation

This study used data from the BRHS, which is a large scale population-based cohort of older men, rather than an institutionalized older population. The magnitude of associations between temperature and sedentary time were not materially affected by excluding men who were depressed or/and with mobility limitations. Thanks to accelerometers it was possible to overcome problems of recall error, which is known to be more common in older individuals.^21^ Therefore, an objective measure is more accurate and recommended, considering the proportion of time older adults spent in sedentary behaviors.^22^ Moreover, we corroborated previous findings which have investigated accelerometer-measured physical activity outcomes: as in earlier studies we showed that low maximum temperatures, fewer hours of sunshine, and higher relative humidity were associated with fewer steps per day, and less time spent in LIPA and MVPA.^5^,^6^ We also demonstrated that the association of maximum temperature with physical activity was strongest in comparison with associations of sunshine duration and humidity with physical activity. Our findings suggested that maximum temperature is the most important predictor of physical activity in the UK. However, earlier studies which took place in Germany, Scotland and Japan had identified a range of different meteorological factors as being the most important, such as global radiation,^23^ day length and diurnal minimum temperature,^5^ rainfall and mean temperature.^24^,^25^ However, we would expect that, as in our results, radiation and other temperature variables are positively correlated with maximum temperature.

The study have some limitations: men who did not accept our invitation to participate in the study were about 2 years older and had higher BMI measured 10 years earlier; implying that overall physical activity (eg, total number of steps) might be lower in the general population. Our study is also limited by studying almost exclusively white European older men, who would be expected to spend more time in sedentary behavior, compared with younger individuals.^4^ Moreover, our results may not be generalizable to women, or to other ethnic groups.^26^

We defined sedentary behavior based solely on intensity, rather than intensity and posture (more widely used), as this study did not aim to investigate the “type” of sedentary behaviors (eg, sitting at a computer, lying on the couch, driving, etc). However, the activity monitors we used provide useful estimates of sedentary time, as they have minimal bias in comparison with other devices able to detect intensity, position and posture.^27^ The importance of position and posture is widely recognized and future studies could further investigate the particular types of sedentary behaviors (eg, watching TV) carried out during the lowest peaks of activity.

Also, during the study period maximum temperatures never reached levels above 30°C. At those high temperatures, more typical of warmer climate zones than the UK, sedentary time may start to increase. During heat waves local authorities tend to alert older individuals, who are usually asked to remain indoors in the heat of the day, to get some rest and sit when necessary, and not engage in strenuous activities.

### Implications

The results may have important implications for guidelines. The UK recommendations suggest that older adults should aim to minimize the time they spend being sedentary each day.^8^ Our findings provided more justification for minimizing sedentary behaviors particularly at low temperatures, a typical element of the winter season. Replacing some of the time spent in sedentary behaviors into more active behaviors may have beneficial effects on health. However, to find ways to reduce sedentariness is challenging, as in modern life opportunities for sedentary behaviors are everywhere. To date, findings from the ProActive65+ trial suggested that older adults with poor self-rated health, higher BMI and history of smoking are more likely to reduce the sedentary time from an exercise intervention.^28^ On the other hand, it is likely that interventions targeting individuals’ psychological and environmental barriers (beliefs, feelings, and perspectives on participations in physical activity) may be a valid alternative for replacing sedentary time with more active behaviors.^29^,^30^ Providing recommendations for simple do-it-yourself exercises (eg, standing up or walking while watching TV, toe rises, calf and chest stretching) could be helpful. In older individuals, simple targets can make the reduction in sedentary behavior easier to achieve and relevant on a daily basis.^31^ Also, providing physically and economically accessible indoor opportunities for promoting more active behaviors during winter should be encouraged.

The temperature-related variation in sedentary time observed in this study could be relevant to the temperature-related variation in mortality risk.^32^ It is plausible that persisting low temperatures in winter (primary determinant) may be a contributing factor which increases the sedentary time, as well as other risk factors levels (eg, inflammatory markers, such as C-Reactive Protein and Interleukin-6^33^) contributing to the excess of winter mortality.^34^ We estimated an increase of 26 minutes in sedentary time at lower versus higher temperatures. According to previous studies in older adults, replacing 30 minutes of sedentary time with light physical activity was independently associated with a significant reduction in mortality risk (HR = 0.80).^35^ However, future investigations are needed to establish how temperature-related variations in sedentary time may contribute to the temperature-related variations in mortality risk.

## Conclusions

In this study of older adults, we demonstrated that sedentary time increased at lower maximum temperatures. These findings are relevant for guidelines: interventions may consider targeting older adults in winter, when temperatures are lower, providing recommendations for minimizing sedentariness on a daily basis.

## Supplementary Material

Supp file

## Figures and Tables

**Table 1:** Adjusted Associations Between Quintiles (Q) of Maximum Temperature and Sedentary Time (n = 1361)^a^

Quintiles of maximum temperature (°C)	Mean difference (95% CI) in sedentary time (minutes per day)
5Q (19.1; 29.5)	Reference
4Q (16.6; 19.0)	+7 (3; 11)
3Q (13.1; 16.5)	+14 (10; 19)
2Q (9.3; 13.0)	+21 (15; 27)
1Q (–3.5; 9.2)	+26 (19; 33)

a Multilevel regression models (level 1 = date, level 2= individual) adjusted for age, social class, BMI, chronic conditions, mobility limitations, geriatric depression scale, vision problems, smoking status, daily wear time, day of the week, wear day order, and day length.

*Note. P*-value for trend < 0.001.
