# Supplementary material for: Association of Maximum Temperature With Sedentary Time in Older British Men
Source: J Phys Act Health. Author manuscript; Available in PMC 2019 Jan 3. (PMC6317701; doi:10.1123/jpah.2016-0468)
Supplement: Supp file [file NIHMS80488-supplement-Supp_file.docx]

**Appendix S1**

*Objectively measured sedentary behaviour and physical activity assessment*

All men who attended an assessment at their local primary care centre were asked to wear the Actigraph GT3x accelerometer (Pensacola, Florida) over the right hip on an elasticated belt for 7 days, during waking hours, removing it only for bathing, swimming or showering and returning the device by post. The study participants wore the accelerometer in between May 2010 and July 2012. The total number of men (by month) who wore the accelerometer was: n=93(6.8%) in January, n=145(10.7%) in February, n=150(11.0%) in March, n=30(2.2%) in April, n=115(8.5%) in May, n=121(8.9%) in June, n=152(11.2%) in July, n=43(3.2%) in August, n=150(11.0%) in September, n=170(12.5%) in October, n=166(12.2%) in November, 26=1.9%) in December. Actigraph accelerometers record physical activity “counts” and steps, which both depend upon the frequency and intensity of the raw acceleration. First, to separate non-wear time from wear time, a sensitivity analysis was carried out using 3 different algorithms (‘non-wear time windows’ of 120, 90 and 60 minutes of zero counts) in a sample of 100 randomly selected men. We compared the self-reported wear time (when the men reported putting on and taking off the accelerometer) to the wear time derived from algorithms using 3 different non-wear time windows. The difference between self-report and algorithm wear time was -8, -1 and +28 minutes for non-wear time window of 120, 90 and 60 minutes respectively. The algorithm which made use of the non-wear time window of 90 minutes performed best; therefore we used that option for the overall population. In detail, non-wear time was identified and excluded using the R package “Physical Activity” [[24](#_ENREF_24)], based on (i) periods of continuous zero activity lasting more than 90 minutes or (ii) periods of zero activity lasting more than 90 minutes broken only by non-zero counts lasting up to 2 minutes, provided no activity counts were detected during both the 30 minutes before and after that interval [[3](#_ENREF_3)]. Valid wear days were defined as >=600 minutes wear time, and participants with at least 3 valid days were included in analyses, a conventional requirement for estimating usual PA level [[25](#_ENREF_25)]. The number of minutes per day in spent in sedentary behaviour, light physical activity (LIPA) and moderate to vigorous physical activity (MVPA) was also derived and categorised using count-based intensity threshold values of counts per minute (CPM) developed for older adults, as in previous studies; the cut-points used were <100, 100-1040, >1040 CPM for sedentary time (<1.5 METs Metabolic Equivalent of Task), time spent in LIPA (<1.5-2.9 METs) and MVPA (>=3 METs) respectively [[3](#_ENREF_3), [26](#_ENREF_26), [27](#_ENREF_27)].

*Meteorological factors*Meteorological data for each day the men wore the accelerometer were taken from 35 weather stations via the United Kingdom (UK) Meteorological Office network (see Supplementary Figure 1 below). The participants resident in 24 UK towns were matched with the closest weather station via post code of residence (mean distance approximately 10 kilometres). The Meteorological Office provided daily temperatures (maximum and minimum between 09:00 h and 21:00 h), daily hours of sunshine (between 00:00 h and 23:59 h), and relative humidity (at 9am). Additionally, day length was provided and measured as hours of light during the day (from sunrise to sunset). Not all weather stations covered the entire follow-up period, so the few missing temperature data (<1.5%) were imputed using the nearest stations’ values and linear regression modelling, as in previous studies [[28](#_ENREF_28)].

**Supplementary Figure 1** – United Kingdom map which indicates the 24 BRHS towns locations (black circles), and 35 MET stations locations (red triangles)

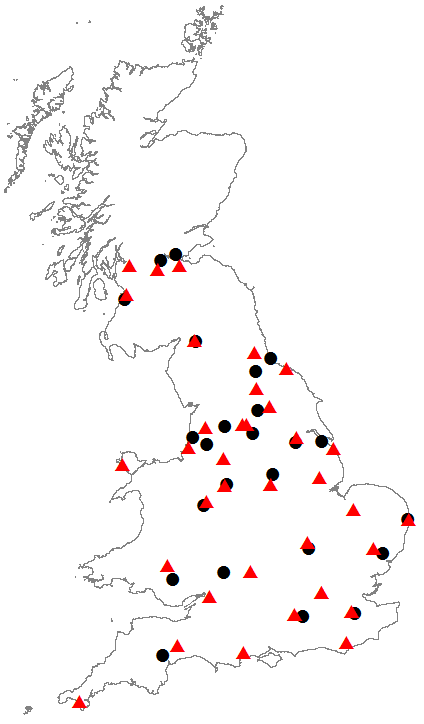


**eTable 1 – Adjusted associations of meteorological factors with sedentary time and physical activity levels. All estimates are reported as mean difference in the outcome levels per a decrease in 1 standard deviation in meteorological factors levels**

| Meteorological factor ^1^ | Outcome | mean difference (95% CI) ^2^ |
| --- | --- | --- |
| MAX temperature (°C) | Sedentary time ^3^ | 11(8,13) |
| MIN temperature (°C) |  | 2(-1,5) |
| Sunshine duration (hours) |  | 9(8,11) |
| Relative Humidity (%) |  | -6(-8,-4) |
| MAX temperature (°C) | Time spent in LIPA ^4^ | -7(-9,-5) |
| MIN temperature (°C) |  | -1(-4,1) |
| Sunshine duration (hours) |  | -6(-7,-5) |
| Relative Humidity (%) |  | 4(3,5) |
| MAX temperature (°C) | Time spent in MVPA ^5^ | -4(-5,-2) |
| MIN temperature (°C) |  | -1(-2,1) |
| Sunshine duration (hours) |  | -3(-4,-2) |
| Relative Humidity (%) |  | 2(1,3) |
| MAX temperature (°C) | Number of steps | -323(-428,-218) |
| MIN temperature (°C) |  | -63(-166,41) |
| Sunshine duration (hours) |  | -270(-326,-213) |
| Relative Humidity (%) |  | 187(122,251) |

^1^ For maximum temperature, minimum temperature, sunshine duration, and relative humidity the standard deviation is 5.8, 5.3, 3.7, and 13.2 respectively. Maximum temperature is defined as the highest air temperatures of the day (from 9am to 9pm); Minimum temperature is the lowest air temperatures of the day (from 9am to 9pm); Relative humidity is a single value recorded every day at 9am; Hours of sunshine were recorded from 00:00 - 23:59 of each day. Pearson correlations (p<0.001) of maximum temperature with minimum temperature is r=0.93, of maximum temperature with sunshine duration is r=0.45, of maximum temperature with relative humidity is r=-0.43.

^2^ Multilevel regression models (level 1=date, level 2= individual) adjusted for age, social class, BMI, chronic conditions, mobility limitations, geriatric depression scale, vision problems, smoking status, daily wear time, day of the week, wear day order, and day length

^3^ Sedentary time is at least one minute where the accelerometer registers values <100cpm
^4^ Time spent in Light physical activity (LIPA) is at least one minute where the accelerometer registers values between 100-1040cpm
^5^ Time spent in Moderate to vigorous physical activity (MVPA) is at least one minute where the accelerometer registers values over 1040 CPM

**eTable 2 – Adjusted associations between quintiles (Q) of maximum temperature and sedentary time in the (i) overall population (n=1361); (ii) excluding participants with depression (n=1071); (iii) excluding participants with any mobility limitations (n=887), excluding participants with depression and with any mobility limitations (n=771).**

|  | Model 1  All, n=1361 | Model 2  Excluding men with depression, n=1071 | Model 3  Excluding men with mobility limitations, n=887 | Model 4  Excluding men with depression and with mobility limitations, n=771 |
| --- | --- | --- | --- | --- |
| Quintiles of maximum temperature (°C) | Mean difference (95%CI) in sedentary time (minutes per day) | Mean difference (95%CI) in sedentary time (minutes per day) | Mean difference (95%CI) in sedentary time (minutes per day) | Mean difference (95%CI) in sedentary time (minutes per day) |
| 5Q (19.1; 29.5), reference | - | - | - | - |
| 4Q (16.6; 19.0) | +7 (3; 11) | +5 (1; 10) | +8 (3; 14) | +8 (2; 13) |
| 3Q (13.1; 16.5) | +14 (10; 19) | +13 (8; 19) | +18 (12; 24) | +18 (11; 25) |
| 2Q (9.3; 13.0) | +21 (15; 27) | +20 (13; 26) | +24 (17; 32) | +22 (14; 30) |
| 1Q (-3.5; 9.2) | +26 (19; 33) | +24 (15; 32) | +28 (19; 37) | +25 (14; 35) |

Model 1: Multilevel regression models (level 1=date, level 2= individual) adjusted for age, social class, BMI, chronic conditions, mobility limitations, geriatric depression scale, vision problems, smoking status, daily wear time, day of the week, wear day order, and day length

Model 2 = adjusted as Model 1 but omitting geriatric depression scale

Model 3 = adjusted as Model 1 but omitting mobility limitations

Model 4 = adjusted as Model 1 but omitting geriatric depression scale and mobility limitations
